# Bayesian phylogenetic and phylodynamic data integration using BEAST 1.10

**DOI:** 10.1093/ve/vey016

**Published:** 2018-06-08

**Authors:** Marc A Suchard, Philippe Lemey, Guy Baele, Daniel L Ayres, Alexei J Drummond, Andrew Rambaut

**Affiliations:** 1 Department of Biomathematics, David Geffen School of MedicineUniversity of California, Los Angeles, 621 Charles E. Young Dr., South, Los Angeles, CA, 90095 USA; 2 Department of Biostatistics, Fielding School of Public HealthUniversity of California, Los Angeles, 650 Charles E, Young Dr., South, Los Angeles, CA, 90095 USA; 3 Department of Human Genetics, David Geffen School of Medicine, University of California, Los Angeles, 695 Charles E. Young Dr., South, Los Angeles, CA, 90095 USA; 4 Department of Microbiology and Immunology, Rega Institute, KU Leuven, Herestraat 49, 3000 Leuven, Belgium; 5 Center for Bioinformatics and Computational Biology, University of Maryland, College Park, 125 Biomolecular Science Bldg #296, College Park, MD 20742 USA; 6 Department of Computer Science, University of Auckland, 303/38 Princes St., Auckland, 1010 NZ; 7 Centre for Computational Evolution, University of Auckland, 303/38 Princes St., Auckland, 1010 NZ; 8 Institute of Evolutionary Biology, University of Edinburgh, Ashworth Laboratories, Edinburgh, EH9 3FL UK

**Keywords:** phylogenetics, phylodynamics, Bayesian inference, Markov chain Monte Carlo

## Abstract

The Bayesian Evolutionary Analysis by Sampling Trees (BEAST) software package has become a primary tool for Bayesian phylogenetic and phylodynamic inference from genetic sequence data. BEAST unifies molecular phylogenetic reconstruction with complex discrete and continuous trait evolution, divergence-time dating, and coalescent demographic models in an efficient statistical inference engine using Markov chain Monte Carlo integration. A convenient, cross-platform, graphical user interface allows the flexible construction of complex evolutionary analyses.

## 1. Introduction

First released over 14 years ago, the Bayesian Evolutionary Analysis by Sampling Trees (BEAST) software package has become firmly established in a broad diversity of biological fields from phylogenetics and paleontology, population dynamics, ancient DNA, and the phylodynamics and molecular epidemiology of infectious disease (Drummond et al. 2012). BEAST's specific focus on time-scaled trees, and the evolutionary analyses dependent on them, has given it a unique place in the toolbox of molecular evolution and phylogenetic researchers. Since inception, a strong motivation for BEAST development has been the rapid growth of pathogen genome sequencing as part of public health responses to infectious diseases (Grenfell et al. 2004). In particular, fast evolving viruses can now be tracked in near real-time (see, e.g. Quick et al. 2016) to understand their epidemiology and evolutionary dynamics.

In BEAST version 1.10, we have introduced a series of advances with a particular focus on delivering accurate and informative insights for infectious disease research through the integration of diverse data sources, including phenotypic and epidemiological information, with molecular evolutionary models. These advances fall into three broad themes—the integration of diverse sources of extrinsic information as covariates of evolutionary processes, the increased flexibility and modularization of the model design process with robust and accurate model testing methods, and substantial improvements on the speed and efficiency of the statistical inference.

## 2. Data integration

Many traits in phylogenetics are represented as or partitioned into a finite number of discrete values, with geographical location standing out as a popular example. Because BEAST is dedicated to sampling time-scaled phylogenies, new developments of discrete character mapping enable the reconstruction of timed viral dispersal patterns while accommodating phylogenetic uncertainty. By extending the discrete diffusion models to incorporate empirical data as covariates or predictors of transition rates, BEAST can simultaneously test and quantify a range of potential predictive variables of the diffusion process (Lemey et al. 2014). Further, realizations of the trait transition process can also be efficiently produced, to pinpoint the nature and timing of changes in evolutionary history beyond ancestral node state reconstruction (termed Markov jumps), or to infer the time spent in a particular state (Markov rewards) (Minin and Suchard 2008). For molecular data, fast stochastic mapping approaches are also employed to obtain site-specific $d_{N}/d_{S}$ estimates, integrating over the posterior distribution of phylogenies and ancestral reconstructions to quantify uncertainty on these measures of the selective forces on individual codons (Lemey et al. 2012).

Multivariate continuous traits are incorporated using phylogenetic Brownian diffusion processes, modelling the shared ancestral dependence across taxa and the correlations between these variables. Such continuous models have most frequently been applied to diffusion on a geographical landscape with the traits representing coordinates and the phylogeny reconstructing the epidemiological process within the host population (Lemey et al. 2010). The landscapes can also represent other spaces, and integration of antibody binding assay data have extended ‘antigenic cartography’ (Smith et al. 2004) approaches to model simultaneous antigenic and genetic evolution and infer the viral trajectories in the immunological space generated by the host population (Bedford et al. 2014).

Standard Brownian diffusion processes that assume a zero-mean displacement along each branch may however be unrealistic for many evolutionary problems (including geographical reconstruction). A recently developed relaxed directional random walk allows the diffusion processes to take on different directional trends in different parts of the phylogeny while preserving model identifiability (Gill et al. 2017) and opens up these processes for a wide range of applications. BEAST 1.10 also extends multivariate phylogenetic diffusion to latent liability model formulations in order to assess correlations between traits of different data types, including (various combinations of) continuous, binary and discrete traits (Cybis et al. 2015), as demonstrated by applications to flower morphology, antibiotic resistance, and viral epitope evolution. To infer correlations between high-dimensional traits computationally efficiently, a novel phylogenetic factor analysis approach assumes that a small unknown number of independent evolutionary factors evolve along the phylogeny and generate clusters of dependent traits at the tips (Tolkoff et al. 2018).

Further extending the data integration approach, BEAST 1.10 includes a flexible framework for incorporating time-varying covariates of the effective population size over time. This uses Gaussian Markov random fields to reconstruct smoothed effective population size trajectories while simultaneously estimating to what extent predictor variables (e.g. fluctuations in climatic factors, host mobility, or vector density) may have driven the dynamics (Gill et al. 2016). Using a similar generalized linear modeling (GLM) approach, classical epidemiological time-series data such as case counts (Gill et al. 2016) can be integrated with pathogen genome sequence data to provide joint inference of important epidemiological parameters.

Finally, recent host-transmission models allow the integration of complete or partial knowledge of a pathogen’s transmission history, enabling the simultaneous inference of within-host population dynamics, viral evolutionary processes, and transmission times and bottlenecks (Vrancken et al. 2014). Likewise, other priors enable the reconstruction of transmission trees of infectious disease epidemics and outbreaks, while accommodating phylogenetic uncertainty and employ a newly designed set of phylogenetic tree proposals that respect node partitions (Hall et al. 2015).

## 3. Flexible model design

BEAST's companion graphical user interface program, BEAUti, allows the user to import data, select models, choose prior distributions, and specify the settings for both Bayesian inference and marginal likelihood estimation. Our efforts on BEAUti 1.10 have focused on allowing the user to easily link or unlink substitution, clock and tree models across multiple partitions as well as linking individual parameters to provide considerable adaptability in model design. Additionally, BEAUti can also group various parameters in a hierarchical phylogenetic model prior (Suchard et al. 2003), which allows parameters to take different values but be linked by a common distribution, the parameters of which can then be inferred. For example, flexible codon model parameterizations, using hierarchical phylogenetic models (Baele et al. 2016b) and incorporating a range of potential predictive variables for substitution behaviour (Bielejec et al. 2016a), provide insight into the tempo and mode of pathogen evolution.

Marginal likelihood estimation to compare models using Bayes factors has become common practice in Bayesian phylogenetic inference. BEAST 1.10 now features marginal likelihood estimation (Baele et al. 2012), using path sampling (Gelman and Meng 1998; Lartillot and Philippe 2006) and stepping-stone sampling (Xie et al. 2011), as well as the recently developed generalized stepping-stone sampling (Fan et al. 2011; Baele et al. 2016a) that offers increased accuracy and improved numerical stability by employing the concept of ‘working distributions’, i.e. distributions with known normalizing constants and parameterized using samples from the posterior distribution.

## 4. Performance and efficiency

Increasing model complexity and sequence availability in modern-day analyses have stretched the computational demands of Bayesian phylogenetic inference. To improve efficiency for large-scale sequence data, BEAST 1.10 uses the BEAGLE library (Ayres et al. 2012) that provides access to massive parallelization on a range of computing architectures. In particular, the combination of BEAST 1.10 with BEAGLE 3.0 (Ayres et al., under review) allows multiple data partitions to be parallelized across a single high-performance device (i.e. a GPGPU graphics board) allowing for the utilization of the full capacity of these devices, reducing the computational overheads. As the complexity of phylogenetic model designs increase, concomitant with the surge in scale of genomic data, updating only a parameter associated with a single data partition limits the occupation of the massively multicore devices. To address this we have developed an adaptive multivariate transition kernel that simultaneously updates parameters across all the partitioned data, making more efficient use of available hardware (Baele et al. 2017). Through a combination of these two advances, BEAST 1.10 can yield a sizeable increase in effectively independent posterior samples per unit-time over previous software versions. For the example data described below, we see a 5- to 25-fold improvement depending on the model parameter, using an NVIDIA Titan V.

### 4.1 Example

**Figure 1.**
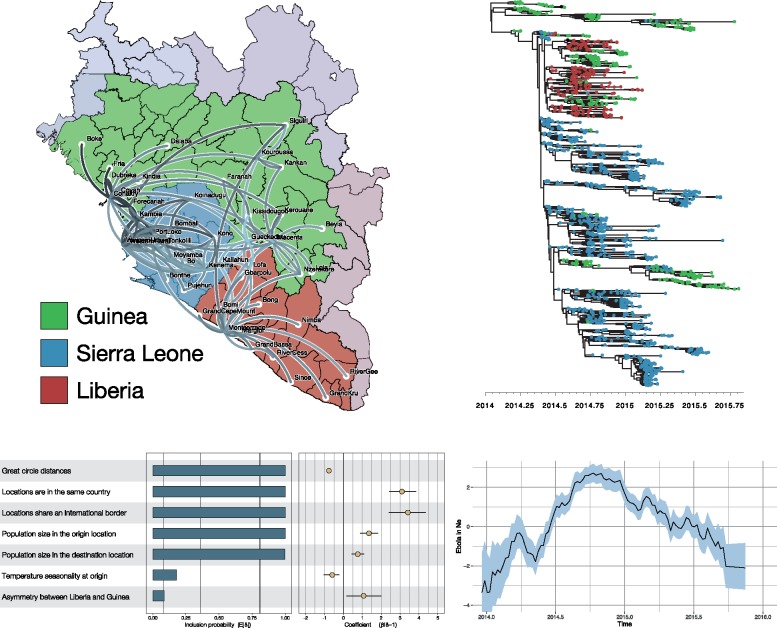
Phylodynamic analysis of the 2013–2016 West African Ebola virus epidemic, encompassing simultaneous estimation of sequence and discrete (geographic) trait data with a GLM fitted to the discrete trait model in order to establish potential predictors of viral transition between locations. Plotted are a snapshot of geographic spread using SpreaD3 (Bielejec et al. 2016b), the maximum clade credibility tree, the posterior estimates of the GLM coefficients for seven possible predictors for Ebola virus spread (Bayes Factor support values of 3, 20, and 150 are indicated by vertical lines) and the effective population size through time, estimated by incorporating case counts.

Figure 1 presents a spatiotemporal reconstruction of Ebola virus evolution and spread during the 2013–2016 West African epidemic, highlighting several aspects of phylodynamic data integration. The estimates are based on a large data set of 1,610 genomes that represent over 5 per cent of the known cases (Dudas et al. 2017). Administrative regions (*n* = 56) are included as discrete sampling locations to estimate viral dispersal through time while testing the contribution of a set of potential covariates to the pattern of spread using a GLM parameterization of phylogeographic diffusion (Lemey et al. 2014). This indicates, for example, the importance of population sizes and geographic distance to explain viral dispersal intensities.

## 5. Relationship to BEAST2 and other software

Distinct from BEAST 1.10 described here, BEAST2 is an independent project (Bouckaert et al. 2014) intended as a platform that more readily facilitates the development of packages of models and analyses by other researchers. Although both projects share many of the same models and the underlying inference framework, BEAST has increasingly focused on the analysis of rapidly evolving pathogens and their evolution and epidemiology. We affirm that BEAST will continue to be developed in parallel to the BEAST2. While these projects share a recent common origin, each now aims to foster complementary research domains.

A range of other software focusing on phylodynamic analyses of fast-evolving pathogens has been described since the last version of BEAST was published. Of particular note are LSD (To et al. 2016), TreeDater (Volz and Frost 2017), and TreeTime (Sagulenko et al. 2018). These programs use least-squares algorithms (LSD) or maximum likelihood inference (TreeDater, TreeTime) and provide rapid analysis on large data sets for a subset of the models that BEAST provides. However, the former program implements very limited phylodynamic models and the latter two programs require a phylogenetic tree, inferred using other software, as input data, conditioning parameter estimates on this single tree.

### 5.1 Availability

BEAST 1.10 is open source under the GNU lesser general public license and available at https://beast-dev.github.io/beast-mcmc for cross-platform compiled programs and https://github.com/beast-dev/beast-mcmc for software development and source code. It requires Java version 1.6 or greater. Documentation, tutorials, and help are available at http://beast.community and many users actively discuss BEAST usage and development in the ‘beast-users’ GoogleGroup discussion group (http://groups.google.com/group/beast-users). We also host an expanding suite of R tools—designed for posterior analyses using BEAST (https://github.com/beast-dev/RBeast).

## References

[vey016-B1] Ayres D. L., Cummings M. P., et al ‘Under review. BEAGLE 3.0: Improved Usability for a High-Performance Computing Library for Statistical Phylogenetics’, Systematic Biology [WorldCat] 10.1093/sysbio/syz020 PMC6802572 31034053

[vey016-B2] Ayres D. L., Darling A., Zwickl D. J., Beerli P., Holder M. T., Lewis P. O., Huelsenbeck J. P., Ronquist F., Swofford D. L., Cummings M. P., Rambaut A., Suchard M. A. (2012) ‘BEAGLE: An Application Programming Interface and High-Performance Computing Library for Statistical Phylogenetics’, Systematic Biology, 61: 170–3. 21963610 10.1093/sysbio/syr100 PMC3243739

[vey016-B3] Baele G., Lemey P., Bedford T., Rambaut A., Suchard M. A., Alekseyenko A. V. (2012) ‘Improving the Accuracy of Demographic and Molecular Clock Model Comparison While Accommodating Phylogenetic Uncertainty’, Molecular Biology and Evolution, 29: 2157–67. 22403239 10.1093/molbev/mss084 PMC3424409

[vey016-B4] Baele G., Lemey P., Rambaut A., Suchard M. A. (2017) ‘Adaptive MCMC in Bayesian Phylogenetics: An Application to Analyzing Partitioned Data in BEAST’, Bioinformatics, 33: 1798–805. 28200071 10.1093/bioinformatics/btx088 PMC6044345

[vey016-B5] Baele G., Lemey P., Suchard M. A. (2016a) ‘Genealogical Working Distributions for Bayesian Model Testing with Phylogenetic Uncertainty’, Systematic Biology, 65: 250–64. 26526428 10.1093/sysbio/syv083 PMC5009437

[vey016-B6] Baele G., Suchard M. A., Bielejec F., Lemey P. (2016b) ‘Bayesian Codon Substitution Modeling to Identify Sources of Pathogen Evolutionary Rate Variation’, Microbial Genomics, 2: e00005. 10.1099/mgen.0.000057 PMC5320644 28348854

[vey016-B7] Bedford T., Suchard M. A.,, Lemey P., Dudas G., Gregory V., Hay A. J., McCauley J. W., Russell C. A., Smith D. J., Rambaut A. (2014) ‘Integrating Influenza Antigenic Dynamics with Molecular Evolution’, eLife, 3: e01914. 24497547 10.7554/eLife.01914 PMC3909918

[vey016-B8] Bielejec F., Baele G., Rodrigo A. G., Suchard M. A., Lemey P. (2016a) ‘Identifying Predictors of Time-Inhomogeneous Viral Evolutionary Processes’, Virus Evolution, 2: vew023. 27774306 10.1093/ve/vew023 PMC5072463

[vey016-B9] Bielejec F., Baele G., Vrancken B., Suchard M. A., Rambaut A., Lemey P. (2016b) ‘SpreaD3: Interactive Visualization of Spatiotemporal History and Trait Evolutionary Processes’, Molecular Biology and Evolution, 33: 2167–9. 27189542 10.1093/molbev/msw082 PMC6398721

[vey016-B10] Bouckaert R., Heled J., Kühnert D., Vaughan T., Wu C.-H., Xie D., Suchard M. A., Rambaut A., Drummond A. J. (2014) ‘BEAST 2: A Software Platform for Bayesian Evolutionary Analysis’, PLoS Computational Biology, 10: e1003537. 24722319 10.1371/journal.pcbi.1003537 PMC3985171

[vey016-B11] Cybis G. B., Sinsheimer J. S., Bedford T., Mather A. E., Lemey P., Suchard M. A. (2015) ‘Assessing Phenotypic Correlation through the Multivariate Phylogenetic Latent Liability Model’, The Annals of Applied Statistics, 9: 969. 27053974 10.1214/15-AOAS821 PMC4820077

[vey016-B12] Drummond A. J., Suchard M. A., Xie D., Rambaut A. (2012) ‘Bayesian Phylogenetics with BEAUti and the BEAST 1.7’, Molecular Biology and Evolution, 29: 1969–73. 22367748 10.1093/molbev/mss075 PMC3408070

[vey016-B13] Dudas G., Carvalho L. M., Bedford T., Tatem A. J., Baele G., Faria N. R., Park D. J., Ladner J. T., Arias A., Asogun D., Bielejec F., Caddy S. L., Cotten M., D’Ambrozio J., Dellicour S., Caro A. D., Diclaro J. D. II, Durrafour S., Elmore M. J., Fakoli L. S. III, Faye O., Gilbert M. L., Gevao S. M., Gire S., Gladden-Young A., Gnirke A., Goba A., Grant D. S., Haagmans B. L., Hiscox J. A., Jah U., Kargbo B., Kugelman J. R., Liu D., Lu J., Malboeuf C. M., Mate S., Matthews D. A., Matranga C. B., Meredith L. W., Qu J., Quick J., Pas S. D., Phan M. V. T., Pollakis G., Reusken C. B., Sanchez-Lockhart M., Schaffner S. F., Schieffelin J. S., Sealfon R. S., Simon-Loriere E., Smits S. L., Stoecker K., Thorne L., Tobin E. A., Vandi M. A., Watson S. J., West K., Whitmer S., Wiley M. R., Winnicki S. M., Wohl S., Wölfel R., Yozwiak N. L., Andersen K. G., Blyden S. O., Bolay F., Carroll M. W., Dahn B., Diallo B., Formenty P., Fraser C., Gao G. F., Garry R. F., Goodfellow I., Günther S., Happi C. T., Holmes E. C., Keïta S., Kellam P., Koopmans M. P. G., Kuhn J. H., Loman N. J., Magassouba N., Naidoo D., Nichol S. T., Nyenswah T., Palacios G., Pybus O. G., Sabeti P. C., Sall A., Ströher U., Wurie I., Suchard M. A., Lemey P., Rambaut A. (2017) ‘Virus Genomes Reveal Factors That Spread and Sustained the Ebola Epidemic’, Nature, 544: 309–15. 28405027 10.1038/nature22040 PMC5712493

[vey016-B14] Fan Y., Wu R., Chen M. H., Kuo L., Lewis P. O. (2011) ‘Choosing among Partition Models in Bayesian Phylogenetics’, Molecular Biology and Evolution, 28: 523–32. 20801907 10.1093/molbev/msq224 PMC3002242

[vey016-B15] Gelman A., Meng X.-L. (1998) ‘Simulating Normalizing Constants: From Importance Sampling to Bridge Sampling to Path Sampling’, Statistical Science, 13: 163–85.

[vey016-B16] Gill M. S., Ho T., Si L., Baele G., Lemey P., Suchard M. A. (2017) ‘A Relaxed Directional Random Walk Model for Phylogenetic Trait Evolution’, Systematic Biology, 66: 299–319. 27798403 10.1093/sysbio/syw093 PMC6075548

[vey016-B17] Gill M. S., Lemey P., Bennett S. N., Biek R., Suchard M. A. (2016) ‘Understanding past Population Dynamics: Bayesian Coalescent-Based Modeling with Covariates’, Systematic Biology, 65: 1041–56. 27368344 10.1093/sysbio/syw050 PMC5066065

[vey016-B18] Grenfell B. T., Pybus O. G., Gog J. R., Wood J. L. N., Daly J. M., Mumford J. A., Holmes E. C. (2004) ‘Unifying the Epidemiological and Evolutionary Dynamics of Pathogens’, Science, 303: 327–32. 14726583 10.1126/science.1090727

[vey016-B19] Hall M., Woolhouse M., Rambaut A. (2015) ‘Epidemic Reconstruction in a Phylogenetics Framework: Transmission Trees as Partitions of the Node Set’, PLoS Computational Biology, 11: e1004613. 26717515 10.1371/journal.pcbi.1004613 PMC4701012

[vey016-B20] Lartillot N., Philippe H. (2006) ‘Computing Bayes Factors Using Thermodynamic Integration’, Systematic Biology, 55: 195–207. 16522570 10.1080/10635150500433722

[vey016-B21] Lemey P., Minin V. N., Bielejec F., Pond S. L. K., Suchard M. A. (2012) ‘A Counting Renaissance: Combining Stochastic Mapping and Empirical Bayes to Quickly Detect Amino Acid Sites under Positive Selection’, Bioinformatics, 28: 3248–56. 23064000 10.1093/bioinformatics/bts580 PMC3579240

[vey016-B22] Lemey P., Rambaut A., Bedford T., Faria N., Bielejec F., Baele G., Russell C. A., Smith D. J., Pybus O. G., Brockmann D. et al (2014) ‘Unifying Viral Genetics and Human Transportation Data to Predict the Global Transmission Dynamics of Human Influenza H3N2’, PLoS Pathogens, 10: e1003932. 24586153 10.1371/journal.ppat.1003932 PMC3930559

[vey016-B23] Lemey P., Rambaut A., Welch J., Suchard M. (2010) ‘Phylogeography Takes a Relaxed Random Walk in Continuous Space and Time’, Molecular Biology and Evolution, 27: 1877–85. 20203288 10.1093/molbev/msq067 PMC2915639

[vey016-B24] Minin V. N., Suchard M. A. (2008) ‘Fast, Accurate and Simulation-Free Stochastic Mapping’, Philosophical Transactions of the Royal Society of London. Series B, Biological Sciences, 363: 3985–95. 18852111 10.1098/rstb.2008.0176 PMC2607419

[vey016-B25] Quick J., Loman N., Duraffour S., Simpson J. et al (2016) ‘Real-Time, Portable Genome Sequencing for Ebola Surveillance’, Nature, 530: 228–32. 26840485 10.1038/nature16996 PMC4817224

[vey016-B26] Sagulenko P., Puller V., Neher R. A. (2018) ‘Treetime: Maximum-Likelihood Phylodynamic Analysis’, Virus Evolution, 4: vex042. 29340210 10.1093/ve/vex042 PMC5758920

[vey016-B27] Smith D. J., Lapedes A. S., de Jong J. C., Bestebroer T. M., Rimmelzwaan G. F., Osterhaus A. D. M. E., Fouchier R. A. M. (2004) ‘Mapping the Antigenic and Genetic Evolution of Influenza Virus’, Science, 305: 371–6. 15218094 10.1126/science.1097211

[vey016-B28] Suchard M. A., Kitchen C. M. R., Sinsheimer J. S., Weiss R. E. (2003) ‘Hierarchical Phylogenetic Models for Analyzing Multipartite Sequence Data’, Systematic Biology, 52: 649–64. 14530132 10.1080/10635150390238879

[vey016-B29] To T.-H., Jung M., Lycett S., Gascuel O. (2016) ‘Fast Dating Using Least-Squares Criteria and Algorithms’, Systematic Biology, 65: 82–97. 26424727 10.1093/sysbio/syv068 PMC4678253

[vey016-B30] Tolkoff M. R., Alfaro M. E., Baele G., Lemey P., Suchard M. A., 2018 ‘Phylogenetic Factor Analysis’, *Systematic Biology*, 67: 384–99. 10.1093/sysbio/syx066 PMC5920329 28950376

[vey016-B31] Volz E., Frost S. (2017) ‘Scalable Relaxed Clock Phylogenetic Dating’, Virus Evolution, 3: vex025.

[vey016-B32] Vrancken B., Rambaut A., Suchard M. A., Drummond A., Baele G., Derdelinckx I., Van Wijngaerden E., Vandamme A.-M., Van Laethem K., Lemey P. (2014) ‘The Genealogical Population Dynamics of HIV-1 in a Large Transmission Chain: Bridging within and among Host Evolutionary Rates’, PLoS Computational Biology, 10: e1003505. 24699231 10.1371/journal.pcbi.1003505 PMC3974631

[vey016-B33] Xie W., Lewis P. O., Fan Y., Kuo L., Chen M. H. (2011) ‘Improving Marginal Likelihood Estimation for Bayesian Phylogenetic Model Selection’, Systematic Biology, 60: 150–60. 21187451 10.1093/sysbio/syq085 PMC3038348

